# Hydrophobic Modification of Polyacrylamides with Oligo(Lactide): A Comparative Study in Two Solvent Systems

**DOI:** 10.1021/acsomega.5c09403

**Published:** 2025-12-29

**Authors:** Larissa Regina Rabaioli, Vanessa Martins Picoli, Augusto Cesar de Carvalho Peres, Cesar Liberato Petzhold

**Affiliations:** † Federal University of Rio Grande do Sul, Av. Bento Gonçalves, 9500, Porto Alegre, Rio Grande do Sul 90650-001, Brazil; ‡ Pontifical Catholic University of Rio de Janeiro, Rua Marquês de São Vicente, 225, Rio de Janeiro, Rio de Janeiro 22451-900, Brazil; § Petróleo Brasileiro S.A, Centro de Pesquisas, Desenvolvimento e Inovação Leopoldo Américo Miguez de Mello (Cenpes), Av. Horácio Macedo, 950—Cidade Universitária, Rio de Janeiro, Rio de Janeiro 21941-915, Brazil

## Abstract

The control and modification of polyacrylamide viscosity through the incorporation of hydrophobic monomers have gained considerable attention due to their cost-effectiveness and potential use in enhanced oil recovery. Hydrophobically modified polyacrylamide copolymers containing oligo­(lactide) macromonomers were synthesized by free radical polymerization in two solvent mixtures: methanol/water and tetrahydrofuran/water. A macromonomer with five repeating units was incorporated at 2, 5, and 10 mol %, as determined by proton nuclear magnetic resonance. Copolymers obtained in tetrahydrofuran/water showed higher incorporation efficiency but lower molar mass than those synthesized in methanol/water, according to size-exclusion chromatography. The latter presented better rheological performance, with higher dynamic viscosities in aqueous solution. Although molar mass affected the results, hydrophobic interactions proved to be the main factor in viscosity enhancement, as homopolymers displayed inferior behavior.

## Graphical abstract

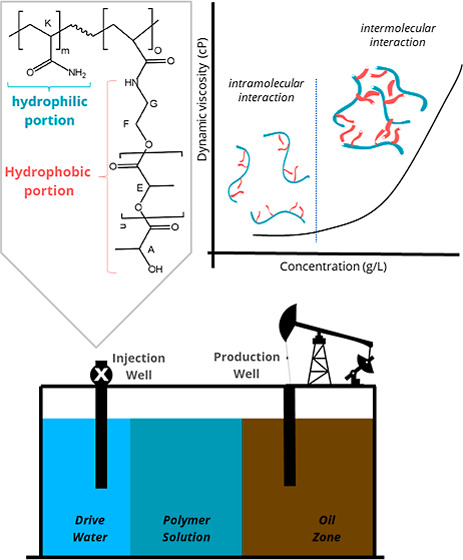

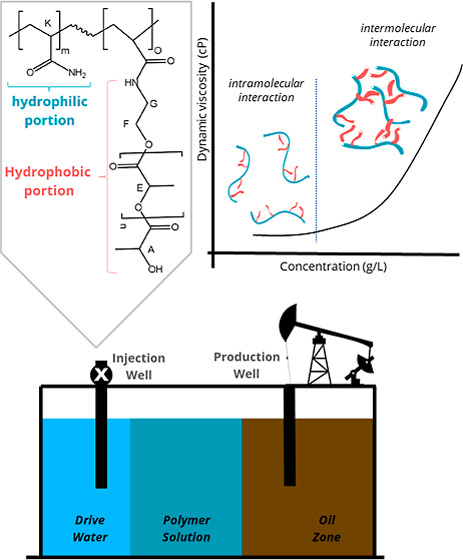

## Introduction

Due to the growing global demand for energy, fossil fuels remain the primary energy source.^1^ In this context, the development of technologies aimed at enhancing oil recovery has gained increasing relevance, since approximately 60% of the oil remains trapped in the reservoir when only primary recovery methods are employed.^2^ Oil extraction typically occurs in three stages: the primary stage, which relies solely on the natural pressure of the reservoir (≈15% recovery); the secondary stage, which involves the injection of water or gas (an additional 10–20% recovery); and the tertiary or enhanced oil recovery (EOR) stage, which can increase recovery by a further 10–35%.^3,4^ It is estimated that 0.3 × 10^12^ m^3^ of conventional oil and 0.8 × 10^12^ m^3^ of heavy oil remain unrecovered using conventional methods.^2^

EOR processes are generally classified as thermal or nonthermal.^5,6^ Among the nonthermal methods, chemical flooding is widely applied following water injection,^2–7^ using polymer, surfactant, or alkaline solutions, with polymer flooding being the most common approach.^2^ In this technique, the polymer solution increases the viscosity of the displacing fluid, thereby improving sweep efficiency and reducing the formation of preferential flow channels within the oil.^8^

The polymers employed can be either synthetic or biopolymers. Xanthan gum is a naturally occurring polysaccharide and a commonly used biopolymer in EOR.^9^ However, it exhibits lower thickening efficiency and is susceptible to degradation.^2^ Conversely, partially hydrolyzed polyacrylamide (HPAM) is the most common synthetic polymer due to its low cost and high water solubility.^2,3,9^ Nevertheless, its application is limited in saline environments owing to the formation of polymer–metal complexes with divalent cations such as Ca^2+^ and Mg^2+^.^8,10^

To overcome these limitations, hydrophobically modified polyacrylamides (HMPAMs) have emerged as promising alternatives, maintaining good performance and enhanced resistance to shear and salinity.^11,12^ These properties arise from the incorporation of hydrophobic groups into the hydrophilic polyacrylamide backbone.^2,6^ Several studies have demonstrated that the rheological behavior of these copolymers depends on both the hydrophobic content and environmental factors such as temperature and salinity.^13–16^

To date, there are no reports on the use of lactide as a hydrophobic macromonomer in EOR applications. Existing studies primarily focus on monomers containing long aliphatic chains or aromatic groups,^17–19^ and only a few investigate ester-containing side groups in nonhydrolyzed polyacrylamide structures.^20,21^

This study investigates the effect of methanol/water (MeOH|H_2_O) and tetrahydrofuran/water (THF|H_2_O) solvent mixtures on the free-radical copolymerization of acrylamide with oligo­(lactide). The use of this oligomer enhances solubility in organic solvents, as the terminal hydroxyl group is more accessible, increasing the overall polarity of the molecule.^22–24^ This behavior is also associated with the lower molar mass of oligo­(lactide) compared to that of poly­(lactide) (PLA) polymers.^22–24^ Macromonomers with approximately five repeating units were copolymerized with acrylamide in different media, with 2, 5, and 10 mol % incorporation levels, to assess synthesis limitations and the influence of incorporation degree on the rheological behavior of the copolymers in aqueous solution.

## Experimental Section

### Materials

Acrylamide was purchased from MP Biomedicals. LS Chemicals supplied sodium chloride (99.9%). Tin­(II) 2-ethylhexanoate (95%), chloroform-*d* (99.8%), deuterium oxide, l-lactide, Hydroquinone and *N*-hydroxyethyl acrylamide (97%) were obtained from Sigma-Aldrich. Sodium bisulfite (58.5%) was acquired from CAQ—Casa da Química (Brazil). Ammonium persulfate (98%) was sourced from Acros Organics. Methanol, tetrahydrofuran, and acetone were purchased from Química Moderna.

### Oligo­(lactide) Macromonomers Synthesis

To obtain hydrophobic segments with five repeating units, two oligo­(lactide) macromonomers (M1 and M2) were synthesized via the ring-opening polymerization (ROP) of l-lactide (LA), as illustrated in Scheme 1a.^23^ The process began with the accurate weighing of recrystallized l-lactide under an inert atmosphere, followed by heating to 130 °C until complete melting.

**Scheme 1:**
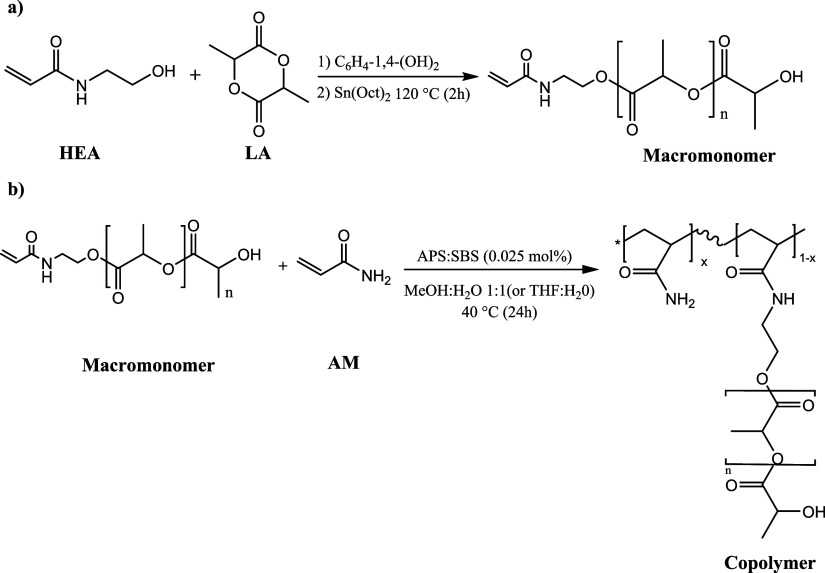
Macromonomer (a) and Copolymer (b) Synthesis

Subsequently, the initiator 2-hydroxyethyl acrylamide (HEA), previously purified through a hydroquinone inhibitor adsorbent column, was added along with the preweighed catalyst stannous octoate (SnOct_2_). The molar ratio between HEA and SnOct_2_ was set at 2.5:0.0025. It is important to note that all reagents were purged with inert gas before contact with l-lactide to prevent moisture interference.

The polymerization reaction was carried out at 130 °C for 2 h under magnetic stirring. The resulting product was stored under refrigeration for subsequent use. According to procedures reported in the literature for the synthesis of similar macromonomers,^23^ no purification steps were performed. Consequently, the crude material was used directly in subsequent reactions to assess its applicability without prior purification. The same approach was adopted for the characterization procedures.

### Synthesis of Copolymers

To evaluate the influence of the solvent in the copolymerization process, copolymers were synthesized via conventional free radical polymerization between M1 (or M2) and the acrylamide (AM) monomer in two different solvent mixtures: tetrahydrofuran and water (THF|H_2_O) and methanol and water (MeOH|H_2_O) (Scheme 1b). It is worth noting that M1 was used in the tetrahydrofuran-containing mixture, while M2 was used in the methanol-containing mixture. In parallel, for comparative purposes, acrylamide homopolymers (PAM) were synthesized under identical reaction conditions for each solvent system (M1-0 and M2-0). Considering the solubility differences between acrylamide and the oligo­(lactide) macromonomer, two experimental approaches were adopted to ensure efficient copolymerization: (I) the use of the aforementioned solvent mixtures (MeOH|H_2_O or THF|H_2_O) during polymerization, to prevent acrylamide precipitation and maintain a homogeneous reaction medium; and (II) the synthesis of copolymers with varying degrees of oligo­(lactide) incorporation (2, 5, and 10 mol %) to validate the suitability of the selected free radical polymerization system for the given oligomer.

### Solvents: THF and H_2_O

Three copolymers with different hydrophobic incorporation levels were synthesized from the crude product of reaction M1: M1-2, containing 2 mol %; M1-5, containing 5 mol %; and M1-10, containing 10 mol %. The synthetic procedure to obtain the three copolymers was identical: a specific amount of M1 was weighed and dissolved in THF, while acrylamide (AM) was weighed and dissolved separately in water. Subsequently, both solutions were mixed in a 1:1 (v/v) ratio, yielding a monomer concentration of 10% (w/v), and maintained under an inert atmosphere for 1 h.

A redox initiation system comprising ammonium persulfate and sodium bisulfite was added afterward, each at 0.025 mol % relative to the monomers. The reaction was conducted at 40 °C for 24 h under magnetic stirring. The polymerization product was dissolved in water and precipitated in acetone to remove unreacted monomers. Following this process, the copolymer was dried in an oven at 60 °C for 48 h, resulting in a white solid.

### Solvents: MeOH and H_2_O

The methodology employed for synthesizing the three copolymers in the MeOH|H_2_O medium was similar to that described for the solvents THF and H_2_O, except that the incorporated macromonomer was the crude product from reaction M2. The oligolactide is first solubilized in MeOH and added to an aqueous solution of acrylamide. As in the previous case, the hydrophobic incorporation level was varied, resulting in M2-2, containing 2 mol %; M2-5, containing 5 mol %; and M2-10, containing 10 mol %.

### Proton Nuclear Magnetic Resonance (^1^H NMR)

The number of repeating units (*n*) and the conversion achieved in reactions M1 and M2 were determined using ^1^H NMR. Spectra were recorded on a Bruker Avance III spectrometer operating at 400 MHz. The crude product of each reaction (M1 and M2) was dissolved in deuterated chloroform (CDCl_3_). For the copolymers, the incorporation content was determined using deuterium oxide (D_2_O) as the solvent. The spectra obtained were analyzed with Mnova software, version 7.1.1, developed by Mestrelab Research.

### Size Exclusion Chromatography (SEC)

The number-average molecular weight (*M*_n_) and dispersity (*D̵*) of the macromonomers and copolymers were determined by size exclusion chromatography (SEC) using a Viscotek system equipped with a GPCmax VE-2001 module and a Malvern Panalytical TDA-402 multidetector. The system was fitted with Shodex columns: KF806L, KF805L, KF804L, and KF803L for organic mode (Mw range: 300 to 2 × 10^3^ kg mol^–1^), and SB802 HQ, SB803 HQ, SB806 HQ, and SB807 HQ (Mw range: 300 to 5 × 10^6^ kg mol^–1^) for aqueous mode, each with an internal diameter (I.D.) of 8.0 mm and a length of 300 mm. Detection was performed using a refractive index (RI) detector. Tetrahydrofuran (THF) was used as the eluent for the macromonomers, whereas a 0.1 M aqueous sodium nitrate (NaNO_3_) solution was employed for the copolymers. Calibration was carried out using a series of narrow-distribution polystyrene standards for organic mode and poly­(2-hydroxyethyl methacrylate) standards for aqueous mode. The calibration curve was constructed by plotting the logarithm of the molecular weight at peak (Log­(*M*_p_)) against the retention volume, following a conventional fifth-order polynomial calibration model. The flow rate was maintained at 1.0 mL min^–1^, with an injection volume of 100 μL, and both the column and detector were held at 45 °C.

### Rheology

The presence of hydrophobic interactions was investigated by comparing the dynamic viscosity measurements between the copolymers and their respective homopolymers, both prepared in aqueous solutions at different polymer concentrations. Rheological analysis was performed using an ARES G2 rheometer (TA Instruments) with a 40 mm cone-and-plate geometry, 1.998° angle, employing a flow ramp method at 30 °C. The shear rate was varied from 0.1 to 300 s^–1^ and the dynamic viscosity determined as the mean value in the region of Newtonian behavior.

## Results and Discussion

### Characterization of Oligo­(lactide) Macromonomers

The macromonomers M1 and M2, used in the copolymer synthesis, were characterized by ^1^H NMR and SEC. To evaluate the chain length of the synthesized oligo­(lactide), ^1^H NMR was employed along with eqs 1 and 2 ^23^ to determine the values of n (number of repeating units) and conversion, respectively. The ^1^H NMR spectrum allowed for clear identification of the material’s structure, even in the absence of purification steps. In Figure 1, the characteristic signals are assigned to the corresponding structural fragments, and the associated chemical shifts are indicated. Additionally, a detailed description of each assigned resonance is provided throughout the text, reinforcing the correlation between the spectrum and the proposed structure.

Repeating⁢ units(n)=(∫E+∫A)/(∫J) 1

2 conversion(%)=(∫E+∫A)/(∫J+∫X+∫A)×100

**Figure 1:**
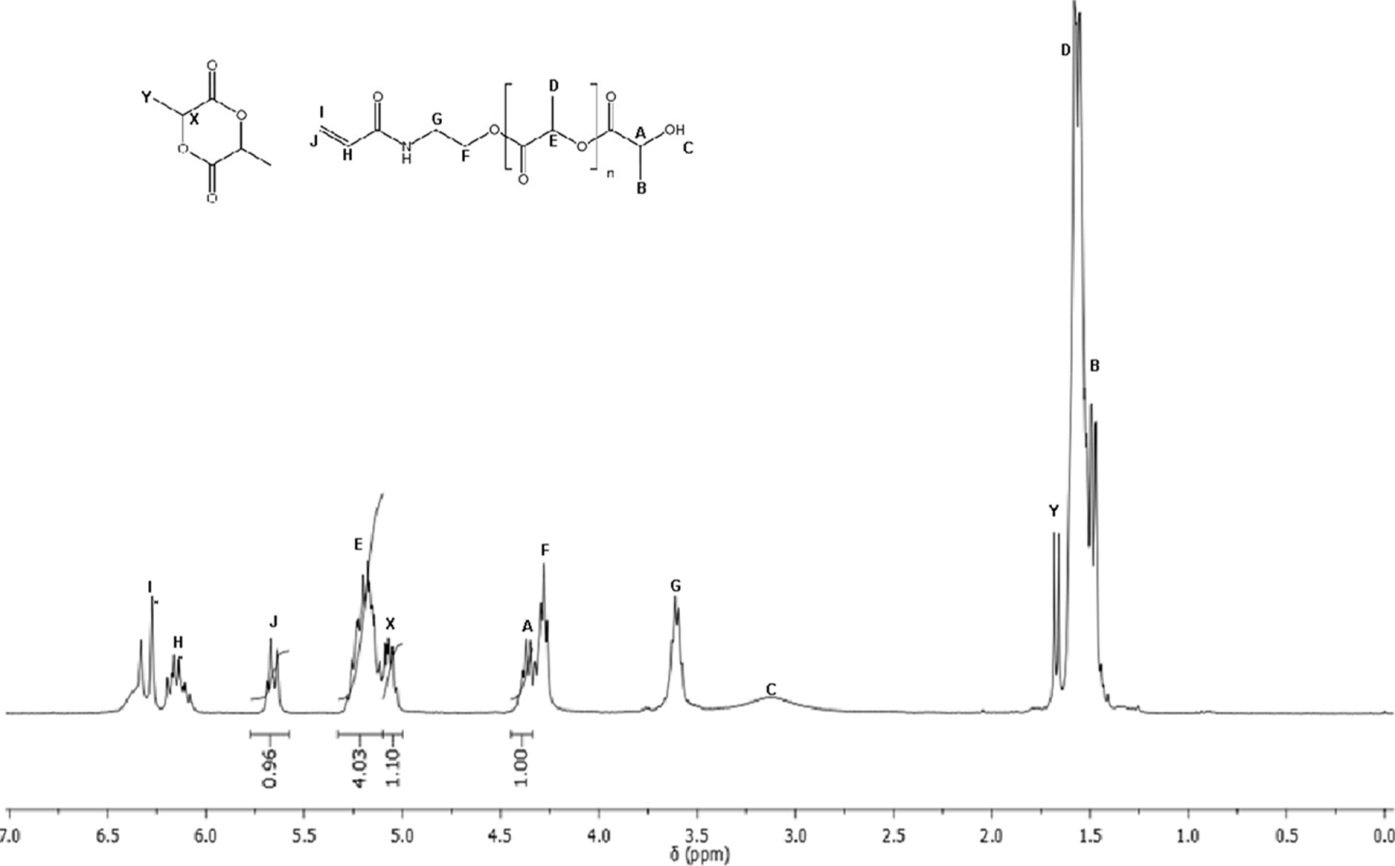
^1^H NMR spectrum (400 MHz) of M2 in CDCl_3_.

As shown in Figure 1, characteristic signals confirming the structure of the macromonomer are observed. The signals between 1.43–1.60 ppm (B, D, and Y) correspond to the aliphatic protons of the CH_2_ and CH_3_ groups along the oligolactide units. The signal at approximately 3.0 ppm (C) is assigned to the terminal hydroxyl proton. The signal at 3.64 ppm (G) refers to the CH_2_ protons adjacent to the amide nitrogen. The signal at 4.30 ppm (F) is attributed to the CH_2_ protons located near the oxygen of the lactic acid unit. The signal at 4.43 ppm (A) corresponds to the terminal methine proton (CH) located at the end of the oligomer chain.

The signal at 5.18 ppm (E) refers to the set of methine protons (CH) present in the repeating lactic acid units. The signals between 5.30 and 6.30 ppm (J and I) correspond to the vinylic protons from the double bond introduced by the macromonomer, with the signal at 6.12 ppm representing the most deshielded vinylic proton, positioned closer to the amide carbonyl group.

As demonstrated in eqs 1 and 2, the calculations of the number of repeating units (*n*) and the macromonomer conversion consider signals E and A, as previously described, as well as signal J at 5.66 ppm, corresponding to the vinylic proton originating from HEA, and the signal at 5.07 ppm, assigned to the methine protons (CH) of the unreacted cyclic l-lactide monomer. The data regarding the values of n and conversion for M1 and M2 are summarized in Table 1.

**Table 1:** Experimental Data Obtained for the Macromonomers M1 and M2^c^

reactions	*n* ^Theo.^	*n* ^Exp.^	conversion (%)^a^	*M*_n_ (g mol-1)^b^	*D̵* ^b^
M1	5	4.2	83	651	1.2
M2	5	4.8	80	462	1.3

a ^1^H NMR.

b SEC.

c ^Theo^Theoretical units ^Exp^Experimental units.

The ring-opening polymerization (ROP) methodology, employing 2-hydroxyethyl acrylamide (HEA) as initiator and l-lactide (LA) as cyclic monomer, proved effective in controlling the average chain length of the hydrophobic segment in both macromonomers, allowing the experimental values of *n* to approach the theoretically predicted ones. The conversion rates for M1 and M2 were approximately 80%, consistent with values reported in the literature for macromonomers synthesized using different co-initiators and the Sn­(Oct)_2_ catalyst.^23–25^ These results demonstrate that the use of a co-initiator system containing HEA in the presence of LA promotes an efficient polymerization reaction, resulting in a low residual monomer concentration in the reaction medium. This feature enables the direct use of the crude macromonomers in subsequent steps without the need for prior purification.

The number-average molar mass (*M*_n_) and dispersity (*D̵* **)** achieved for M1 and M2 were evaluated by SEC. The chromatograms (Supporting Information Figure S1) exhibit multimodal curves for both reactions, a characteristic feature of oligomeric structures composed of units of varying sizes.^23,24,26^ According to the literature, signals observed at lower retention volumes are predominantly associated with an increase in the chain length corresponding to the hydrophobic segment of the macromonomer.^23^ This behavior is consistent, as this molecular region represents the largest structural extension, directly influencing its elution time in chromatographic analyses.

Size exclusion chromatography (SEC) proved suitable for determining the number-average molar mass (*M*_n_) of the macromonomers M1 and M2, given that the experimental values obtained approached the theoretical Mn (475 g·mol^–1^), calculated based on the molar mass of the repeating unit, the degree of polymerization, and the molar mass of the initiator. However, it was observed that the *M*_n_ of M1 is higher than that of M2, which may be attributed to intrinsic limitations of the SEC technique, such as differences in the interactions between the samples and the column or the eluent, which can influence the elution behavior of structures synthesized under identical conditions. Figure S1, presented in the Supporting Information, highlights these structural differences, showing that M1 elutes slightly earlier than M2, suggesting variations in interaction behavior with the chromatographic system, such as the column and the elution solvent.^27^ After all, this result further supports the advantages of the employed synthesis route since ring-opening polymerization (ROP) via a coordination–insertion mechanism shows a lower tendency to side reactions such as racemization and transesterification.^28^

Additionally, the efficient reaction control in the synthesis of M1 and M2 is corroborated by the dispersity (*D̵*) values presented in Table 1, which indicate the formation of polymer chains with narrow molecular weight distribution, characteristic of monodisperse systems.^22,25,31^

### ^1^H NMR and SEC of Copolymers

The copolymers were obtained via free radical polymerization. For the initiator system, a redox pair consisting of ammonium persulfate (initiator) and sodium bisulfite (inorganic reductant) was employed to generate a radical anion capable of initiating the polymerization at a relatively low temperature, 40 °C.^28^ The temperature of the reaction medium was considered due to the potential depolymerization of the oligo­(lactide), a condition favored at elevated temperatures and low pressures through thermal degradation reactions.^29,30^

The structure obtained in each copolymerization reaction was confirmed by ^1^H NMR, which enabled the determination of both the hydrophobic incorporation and the monomer-to-copolymer conversion based on the spectra and eqs 3 and 4.^23^ As expected, higher oligo­(lactide) content in the copolymer hindered solubility in the ^1^H NMR analysis solvent (D_2_O). Figure 2 shows the ^1^H NMR spectrum of M1-2 synthesized in the MeOH/H_2_O mixture.

3 Incorporation(%)=(∫F/2)/(∫K+∫F/2)×100

$$conversion\;\left(\%\right)=\left(\int \frac{K}{3}-\int region\;5.5\;ppm-\int region\;6.5\;ppm\right)/\left(\int \frac{K}{3}+\int E-\int region\;5.5\;ppm-\int region\;6.5\;ppm\right)\times 100$$ 4

**Figure 2:**
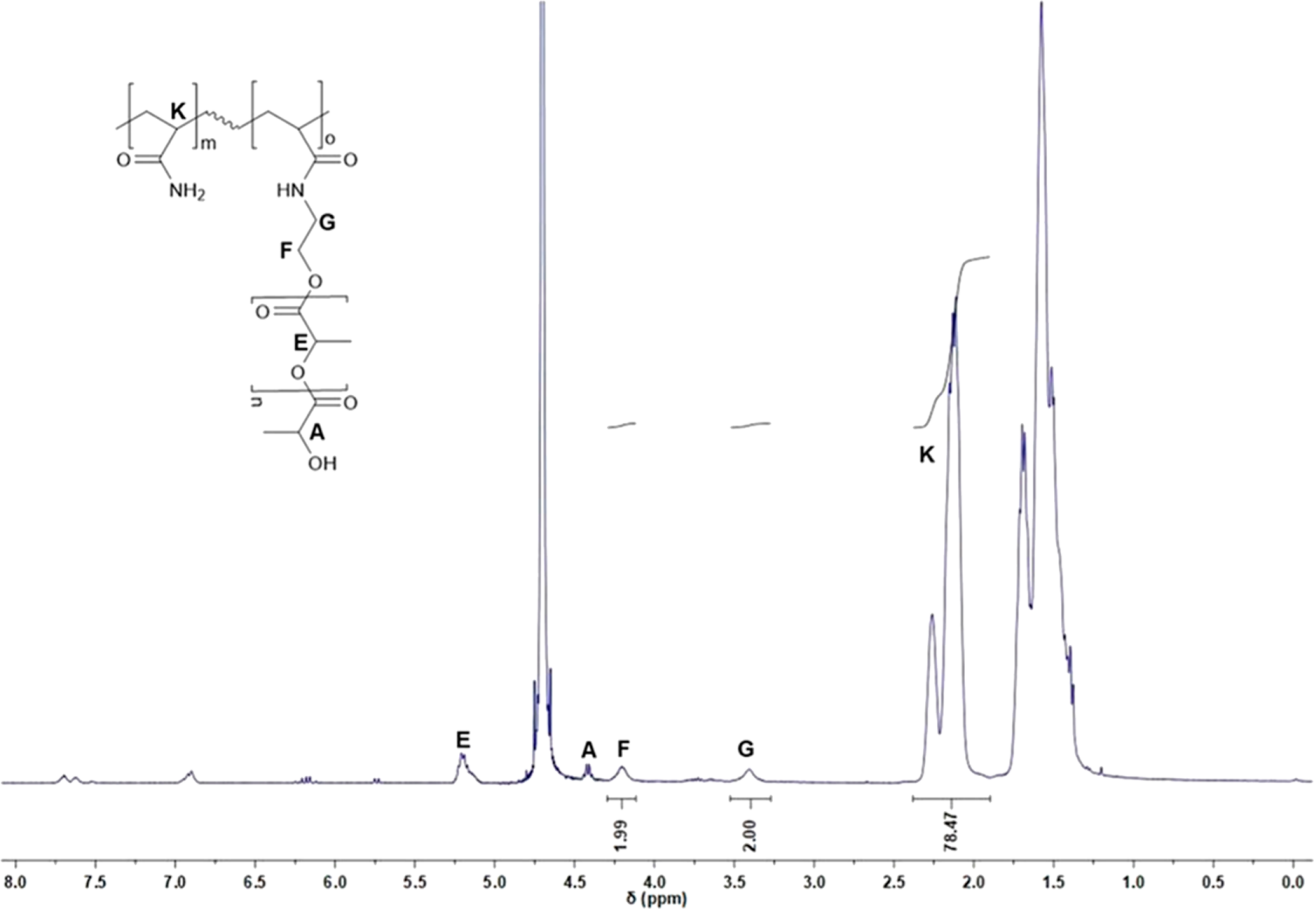
^1^H NMR spectrum (400 MHz) of M1-2 in D_2_O.

As shown in Figure 2, characteristic signals confirming the copolymer structure can be observed. The signals attributed to the acrylamide segment appear in the region K (1.0–2.5 ppm) and correspond to the methylene (CH_2_) and methine (CH) protons. Signals F and G, located at 4.16 and 3.40 ppm, respectively, are assigned to the methylene (CH_2_) protons of the oligo­(lactide) side chain. Signal E, at 5.20 ppm, represents the total methine (CH) protons from the lactic acid units. Signal A (4.43 ppm) corresponds to the terminal methine proton (CH) located at the end of the oligomer chain (as discussed in the previous section). The signals between 5.5 and 6.5 ppm correspond to the vinylic protons of the double bond introduced by the macromonomer, indicating the presence of unreacted macromonomer in the medium.

Based on these diagnostic resonances, which confirm the incorporation of the macromonomer into the polyacrylamide backbone, the integrals of these signals were used in eqs 3 and 4 to calculate the degree of incorporation and monomer conversion.

As identified in the spectrum (Figure 2) and shown in Table 2, the radical polymerization system enabled the incorporation of different amounts of oligo­(lactide) into the polyacrylamide backbone, yielding water-soluble copolymers. The high polarity and the ability to form hydrogen bonds, arising from hydroxyl and ester groups in the oligo­(lactide) structure, facilitate solubility in polar solvents containing hydroxyl groups, which may result in hydrolytic cleavage of the lactic acid segment.^30,31^ In this context, the methine (CH) signals E and A, at 4.42 and 5.20 ppm, respectively, indicate that no hydrolysis of the lactic acid moiety occurred during the copolymerization.

**Table 2:** ^1^H NMR and SEC Data of the Copolymers in Different Solvents^c^

solvents	reaction	conversion (%)^a^	*M*_n_ (g mol^–1^)^b^	*D̵* ^b^	incorporation^Theo^ (mol %)	Incorporation^Exp^ (mol %)
THF and H_2_O	M1-0	100	33,400	4.4	0	0
	M1-2	78	37,600	3.2	2	1.3
	M1-5	65	43,400	3.0	5	3.9
	M1-10	65	39,500	3.2	10	8.1
MeOH and H_2_O	M2-0	89	206,000	3.3	0	0
	M2-2	96	195,000	4.3	2	1.9
	M2-5	78	216,000	4.0	5	1.3
	M2-10	35	190,000	4.0	10	6.5

a Mass conversion.

b SEC.

c ^Theo^Molar ratio of the reagents ^Exp1^H NMR.

The G signal at 3.37 ppm, associated with the methylene (CH_2_) protons from the oligo­(lactide) side chain, further supports that the incorporation of the hydrophobic segment was quantitative in the synthesized copolymers. Additionally, the signals in the 6.5–8.0 ppm range correspond to the protons of the amide group (CONH_2_), confirming the presence of the hydrophilic acrylamide unit.

According to Table 2, an increase in the oligo­(lactide) content leads to lower monomer conversion and reduced experimental incorporation. This trend is observed for both solvent mixtures, being more pronounced in syntheses performed with MeOH. The solubility of the acrylamide monomer must also be considered, as precipitation may occur depending on the medium used. From the M1-0 and M2-0 samples, it is evident that the presence of THF or MeOH does not hinder the formation of acrylamide homopolymer, yielding conversions above 85%.

It is observed that, as the theoretical incorporation content increases, the efficiency of experimental incorporation systematically declines. Regardless of the composition of the reagent mixture employed for monomer solubilization during copolymerization, experimentally determined incorporation never reaches its theoretical maximum. This phenomenon can be attributed to experimental limitations arising from the progressive hydrophobicity of the polymer formed in the reaction medium. As the reaction proceeds, the polymer becomes increasingly hydrophobic relative to the surrounding phase, promoting its precipitation and thereby disrupting the continuity of the copolymerization. This effect likewise manifests in the overall conversion values, which decrease as the proportion of macromonomer incorporation rises. Consequently, experimental incorporation values tend to plateau at specific levels, and achieving higher incorporation contents becomes progressively more difficult under the given reaction conditions.

The number-average molar mass (*M*_n_) and dispersity (*D̵*) (Table 2) of the copolymers were determined by size exclusion chromatography (SEC). The different molar mass values obtained for each copolymer suggest which polymerization solvent is most suitable for the system under investigation, in addition to allowing an assessment of the influence of solubility on the final molar mass. Copolymers synthesized in the THF-containing medium exhibited *M*_n_ values in the order of 10^5^ g mol^–1^, while those synthesized in MeOH presented *M*_n_ values around 10^6^ g mol^–1^. This result reflects the lower ability for chain transfer reactions (Ctr) of methanol (MeOH), whose Ctr value is approximately 0.34, compared with tetrahydrofuran (THF), which has a value around 0.66. These constants, denoted as “Ctr”, quantify the efficiency with which solvent transfers a hydrogen atom to a growing polymer radical, leading to chain termination or transfer and thereby lowering the resulting molar mass.^32^

Furthermore, the role of the initiator system must also be considered, since its concentration directly affects the polymer chain growth and, consequently, the molar mass obtained. In the Supporting Information (Figures S2 and S3), chromatograms of the copolymers and acrylamide homopolymers are presented. For both solvent mixtures, symmetric unimodal curves are observed, indicating the formation of a narrow distribution of structures with similar chain lengths.

### Rheological Study

The effects of hydrophobic modification on the rheological properties of the copolymers were evaluated based on the dynamic viscosity values as a function of polymer solution concentration (Table 3). As demonstrated in the previous section, the copolymers synthesized in the THF|H_2_O system (M1-X) exhibited molar masses 1 order of magnitude lower, even under the same reaction conditions used for copolymers synthesized in MeOH|H_2_O (M2-X). Since viscosity is directly influenced by molar mass, M2-X copolymers displayed higher rheological performance compared to M1-X. However, it is important to note that the main factor contributing to the increase in dynamic viscosity is the hydrophobic moieties. Therefore, copolymers synthesized in the THF-containing mixture are expected to exhibit superior rheological behavior due to their higher degree of hydrophobic incorporation.

**Table 3:** Dynamic Viscosity Data of the Copolymers in Different Solvents (30 °C)^a^

		dynamic viscosity (cP)				
solvents	reaction	2 g L^–1^	10 g L^–1^	20 g L^–1^	50 g L^–1^	100 g L^–1^
THF and H_2_O	M1-0	1.02	1.42	1.96	4.95	17.0
	M1-2	1.08	1.49	2.42	7.24	28.8
	M1-5	0.93	1.35	2.06	5.78	22.1
	M1-10	1.06	1.43	2.07	7.33	38.3
MeOH and H_2_O	M2-0	1.25	4.33	11.0	134	1280
	M2-2	1.24	5.33	18.5	202	1507
	M2-5	1.40	5.74	22.6	316	ND
	M2-10	1.01	9.40	85.0	906	ND

a ND: not determined.

When analyzing each solvent mixture and their respective copolymers individually (Table 3), it can be observed that, from a concentration of 20 g·L^–1^, the increase in hydrodynamic volume in solution is associated with hydrophobic interactions. In both solvent systems, structures containing the oligo­(lactide) macromonomer showed higher dynamic viscosity values than their respective homopolymers. These results confirm that different macromonomer contents induce distinct rheological behaviors.

With a higher hydrophobic content within the hydrophilic backbone, it was initially expected that phase separation might occur, hindering complete solubilization and limiting interactions to intramolecular domains. However, upon evaluating the copolymers with approximately 6–8 mol % incorporation (M1-10 and M2-10), it was observed that these materials presented significantly elevated dynamic viscosity values. This indicates that, above a critical aggregation concentration, intermolecular interactions are promoted, favoring the formation of a three-dimensional network among the hydrophobic segments.^33,34^

From the rheological analysis, it is concluded that the most effective solvent mixture is the one containing MeOH, as it results in the highest dynamic viscosity values. Despite this, since viscosity is directly influenced by molar mass, the methanol-containing mixture demonstrated superior overall performance.

It is important to highlight that the rheological behavior observed in the solvent mixtures resulted in dynamic viscosity values higher than those of the corresponding homopolymers. Compared to copolymers reported in the literature, such as those described by Gouveia and co-workers,^13^ which contain oxygenated groups in their side chains that promote hydrophobic interactions and have incorporation levels similar to those synthesized in this study, the copolymers developed here exhibit superior rheological performance. This is evidenced by the measured molar mass on the order of 10^5^ g mol^–1^, which yields dynamic viscosities comparable to those reported for copolymers with molar masses around 10^6^ g mol^–1^. The enhanced rheological behavior can thus be attributed to the side chains, which promote the formation of an efficient three-dimensional network, enabling high viscosity even at lower molar masses.

## Conclusion

Macromonomers with defined oligo­(lactide) chain lengths were successfully synthesized via ring-opening polymerization of l-lactide with 2-hydroxyethyl acrylamide, requiring no purification due to high conversion and low dispersity. These macromonomers were copolymerized with acrylamide in MeOH|H_2_O and THF|H_2_O solvent mixtures. Copolymers from THF|H_2_O showed incorporation levels close to theoretical values and narrower dispersities, while those from MeOH|H_2_O exhibited higher molar masses (∼10^6^ g mol^–1^). All samples had unimodal molecular weight distributions.

Rheological analysis revealed that increasing polymer concentration led to higher viscosities, especially from 20 g L^–1^ onward, due to hydrophobic interactions among lactic acid segments. Viscosity also increased with higher macromonomer content. All copolymers outperformed the homopolymer, with MeOH|H_2_O systems showing viscosities approaching commercial benchmarks. Further optimization is needed to achieve high viscosities at lower concentrations to improve application efficiency and cost-effectiveness.

## Supplementary Material



## Notes

SEC chromatograms of macromonomers M1 and M2 (Figure S1) and the copolymers synthesized in THF (Figure S2) and in MeOH (Figure S3) (PDF)

## Abbreviations

MeOH: methanol
H_2_O: water
THF: tetrahydrofuran
EOR: enhanced oil recovery
HPAM: partially hydrolyzed polyacrylamide
HMPAM: hydrophobically modified polyacrylamide
ROP: ring-opening polymerization
LA: l-lactide
HEA: 2-hydroxyethyl acrylamide
Sn (Oct_2_): stannous octanoate
^1^H NMR: Proton Nuclear Magnetic Resonance
SEC: Size Exclusion Chromatography
D_2_O: deuterium oxide
CDCl_3_: deuterated chloroform
NaNO_3_: sodium nitrate;
