# Supplementary material for: Hydrophobic Modification of Polyacrylamides with Oligo(Lactide): A Comparative Study in Two Solvent Systems
Source: ACS Omega. 2025 Dec 29;11(1):1999–2006. doi: 10.1021/acsomega.5c09403 (PMC12809852; doi:10.1021/acsomega.5c09403)

Hydrophobic Modification of Polyacrylamides with Oligo(Lactide): A Comparative Study in Two Solvent Systems

Larissa Regina Rabaioli[1], *Vanessa Martins Picoli*[2], *Augusto Cesar de Carvalho Peres*[3], *Cesar Liberato Petzhold*[1*]

[1]Federal University of Rio Grande do Sul, Av. Bento Gonçalves, 9500, Porto Alegre – RS, 90650-001, Brazil

[2]Pontifical Catholic University of Rio de Janeiro- Rua Marquês de São Vicente, 225, Rio de Janeiro - RJ - 22451-900 Brazil

[3] Petróleo Brasileiro S.A. (Petrobras), Leopoldo Américo Miguez de Mello Research, Development and Innovation Center (CENPES), Av. Horácio Macedo, 950, Cidade Universitária, Rio de Janeiro, RJ 21941-915, Brazil

*petzhold@iq.ufrgs.br

SUPPORTING INFORMATION

Figure S1. SEC chromatograms of M1 (blue) and M2 (green) in THF

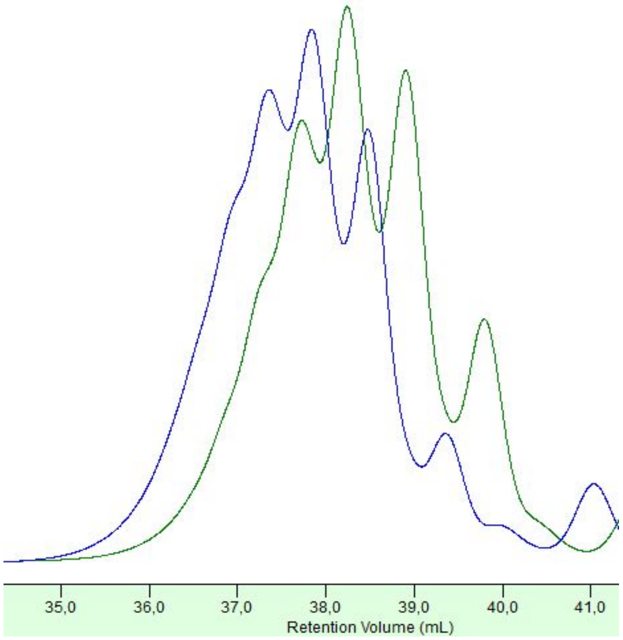

Figure S2. SEC chromatograms of the copolymers synthesized in THF.

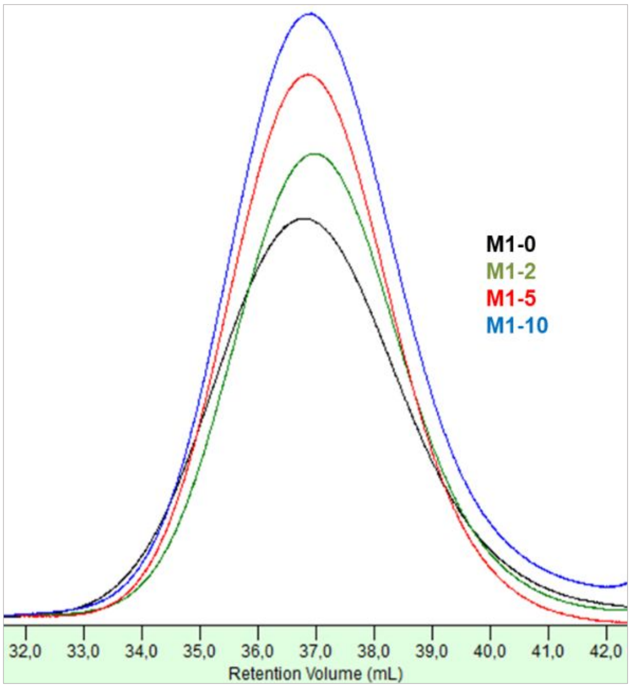

Figure S3. SEC chromatograms of the copolymers synthesized in MeOH.

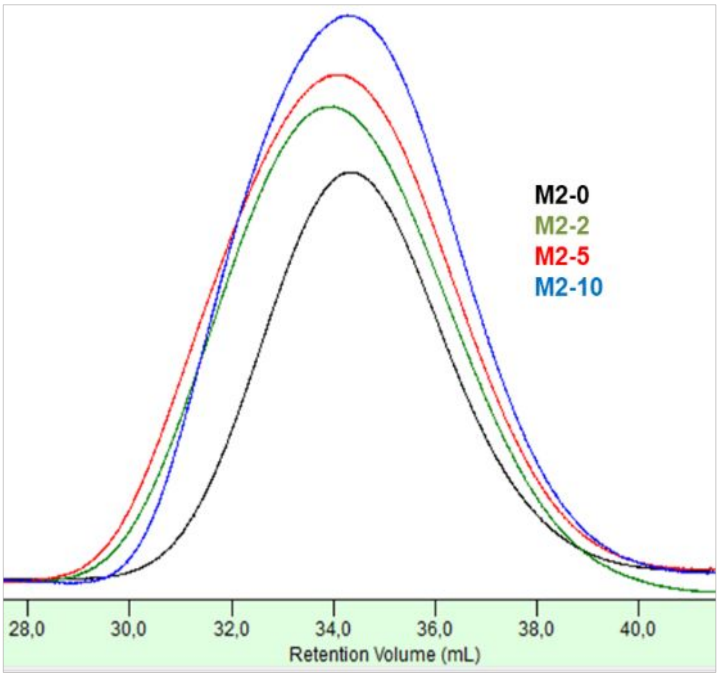

Supplement: Supplementary file 1 [file ao5c09403_si_001.pdf]
